# Comparison of results from tests of association in unrelated individuals with uncollapsed and collapsed sequence variants using tiled regression

**DOI:** 10.1186/1753-6561-5-S9-S15

**Published:** 2011-11-29

**Authors:** Heejong Sung, Yoonhee Kim, Juanliang Cai, Cheryl D Cropp, Claire L Simpson, Qing Li, Brian C Perry, Alexa JM Sorant, Joan E Bailey-Wilson, Alexander F Wilson

**Affiliations:** 1 Genometrics Section, Inherited Disease Research Branch, National Human Genome Research Institute, National Institutes of Health, 333 Cassell Drive, Baltimore, MD 21224, USA; 2 Statistical Genetics Section, Inherited Disease Research Branch, National Human Genome Research Institute, National Institutes of Health, 333 Cassell Drive, Baltimore, MD 21224, USA

## Abstract

Tiled regression is an approach designed to determine the set of independent genetic variants that contribute to the variation of a quantitative trait in the presence of many highly correlated variants. In this study, we evaluate the statistical properties of the tiled regression method using the Genetic Analysis Workshop 17 data in unrelated individuals for traits Q1, Q2, and Q4. To increase the power to detect rare variants, we use two methods to collapse rare variants and compare the results with those from the uncollapsed data. In addition, we compare the tiled regression method to traditional tests of association with and without collapsed rare variants. The results show that collapsing rare variants generally improves the power to detect associations regardless of method, although only variants with the largest allelic effects could be detected. However, for traditional simple linear regression, the average estimated type I error is dependent on the trait and varies by about three orders of magnitude. The estimated type I error rate is stable for tiled regression across traits.

## Background

The assumptions of independence between observations and between independent variables are a major theoretical underpinning of much of traditional statistics. However, because of the linear nature of the genome and the intrinsically familial nature of genetics, these assumptions are often violated when traditional statistical methods are applied to genetic data. As the density of genetic markers increases, ultimately encompassing the entire genome, the correlations between markers increase, depending in large part on the linkage disequilibrium structure in the sample. For unrelated individuals a multiple linear regression approach, including all variants across the entire genome, would be ideal. However, the large number of variants relative to the number of samples and the presence of a large degree of multicollinearity among markers make this approach intractable. In addition, the inclusion of rare sequence variants, including several rare variants in the same gene, makes traditional tests of association problematic because of the low frequency of many of the variants. In this study, we use tiled regression [1] to analyze the unrelated individuals from the simulated Genetic Analysis Workshop 17 (GAW17) data in order to identify the set of variants that are responsible for the variation in quantitative traits and to compare the use of uncollapsed and collapsed sequence variants.

## Methods

### Study population

To examine the data for population substructure, we first performed a principal components analysis on 1,356 common SNPs (minor allele frequency [MAF] > 0.2 and *r*^2^ < 0.2) with Eigensoft, version 3.0 [2]. The self-reported ethnicity of each individual was plotted against the first two principal components. The individuals could be grouped into three populations (Asian, African, and European), except for one self-reported European who was classified in the Asian group. This individual, NA12829, was removed from all subsequent analyses, leaving 696 individuals. However, because the sample sizes for the subpopulations were small, the subpopulations were considered a single sample. We used linear regression to adjust and center each quantitative trait (Q1, Q2, and Q4) for age, sex, and smoking status in each of the 200 replicates.

### Variant coding

We used the genotypes for the 24,473 nonmonomorphic single-nucleotide polymorphisms (SNPs), including common and rare sequence variants (collectively referred to here as sequence variants), as provided (uncollapsed) and with rare sequence variants collapsed [3,4]. To collapse the rare variants, we used two methods: (1) collapsing all variants with a MAF < 0.01 and (2) collapsing nonsynonymous variants with a MAF < 0.01. The rare variants were collapsed into a single variant for each genomic region defined by hot spots (see Tiled regression section). The derived region-wide collapsed variants were coded as the presence or absence of any rare allele within each region. Common variants were left uncollapsed and coded as the number of minor alleles.

### Tiled regression

In tiled regression, the genome is divided into independent segments based on predefined regions. Recombination hot spots (i.e., well-defined regions of increased recombination) are used to delineate regions. The term *tile* denotes both the sequence of DNA between two hot spot regions and a hot spot region itself. Each sequence variant is assigned to a tile based on its physical position. A tile is selected if the multiple linear regression on all variants in the tile shows a significant relationship to trait variation (testing the null hypothesis that all variant coefficients are 0) or if the simple linear regression on any single variant in the tile is significant. A stepwise regression is then used to select the important individual independent variants identified in each selected tile. Thereafter the significant variants are combined across tiles in higher-order stepwise regressions within chromosome and then genome levels. The end result is a multiple linear regression model that includes a set of variants that independently contribute to trait variation. An appropriate null distribution for determining the significance level of the overall results is being investigated, and permutation tests will likely be required to obtain an accurate significance level.

### Test of association for uncollapsed and collapsed sequence variants

We used tiled regression, as implemented in TRAP (Tiled Regression Analysis Package, http://research.nhgri.nih.gov/software/TRAP) [5], to identify the set of independently significant sequence variants that affect each of the covariate-adjusted quantitative traits and to compare results from the uncollapsed and collapsed approaches. Tiles were determined on the basis of the location of recombination hot spots in Human Genome Sequence build 36 [6]. We used critical values of 0.1 and 0.01 for the initial screening of the multiple and simple regressions, respectively. We used a critical value of 0.01 for entering and retaining variables in the stepwise regressions. Simple linear regressions (SLRs) were performed with TRAP and PLINK [7].

We requested the answers for the GAW17 simulated data and compared resulting sets of significant variants to the simulation model to examine power and type I error. We determined measures analogous to average power and type I error rate.

## Results

Results are presented here in detail for uncollapsed variants and for collapsed variants, including only nonsynonymous variants with MAF < 0.01, for the combined populations for the 200 replicates. Results from the analysis of collapsed variants including all variants with MAF < 0.01 were similar to those for the collapsed nonsynonymous variants and are not shown. Results from both SLR implementations (PLINK and the method included in TRAP) were nearly identical, as expected.

**Figure 1:**
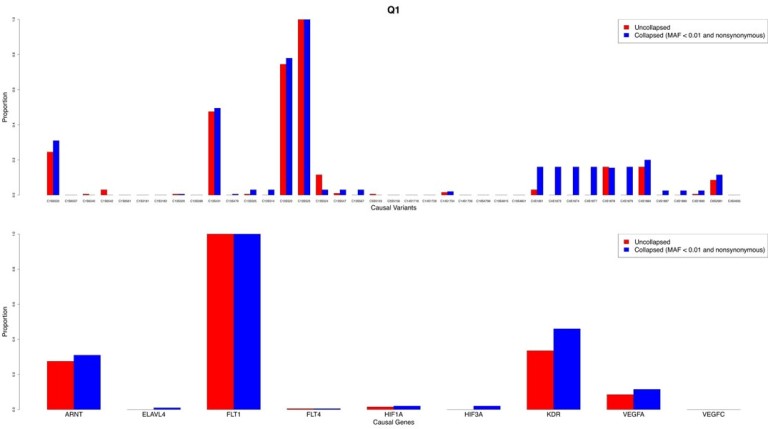
Proportion of 200 replicates identifying each causal variant and gene significant for trait Q1.

**Figure 2:**
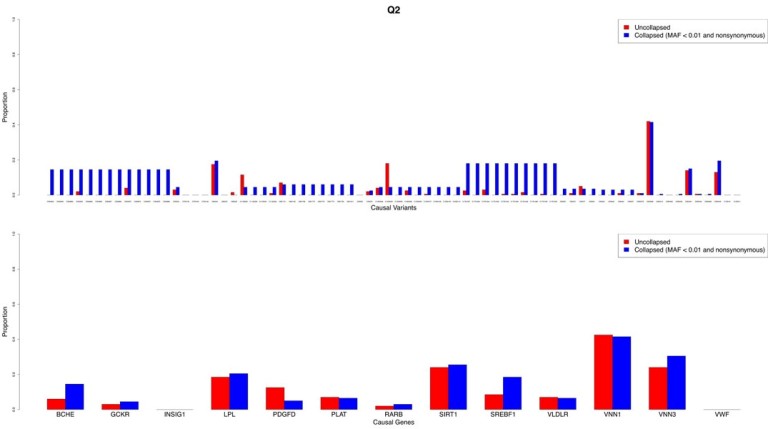
Proportion of 200 replicates identifying each causal variant and gene significant for trait Q2.

Results for traits Q1 and Q2 using the tiled regression method with a critical level of 0.01 are presented in Figures 1 and 2, respectively. The top track illustrates the proportion of the 200 replicates (PoR) that were significant for each causal variant. Similarly, the lower track illustrates the PoR that were significant plotted against each causal gene. Figures 1 and 2 demonstrate that collapsing rare variants generally increases the PoR identifying variants found to be significant, both at the causal variant and gene levels, although this finding appears to be more pronounced for variants with smaller effects. However, for Q1, only variants in *FLT1* and *ARNT* had proportions greater than about 0.3. For Q2, only variants in *VNN1* had a PoR greater than 0.2. Some variants in *VNN3*, *SIRT1*, and *LPL* were identified (PoR < 0.2), but nearly all the other causal variants for Q2 were undetectable.

Table 1 presents the PoR for each of the most frequently found variants in the generating model for traits Q1 and Q2 significant at the 10^−7^ significance level for the SLR and at the 10^−2^ and 10^−7^ critical levels for tiled regression, using the *p*-values generated for the final stepwise regression. Table 1 includes only those variants for which the PoR was greater than 0.2 for any method. This is roughly analogous to the power of the test for each variant, ignoring the identical genotypes across replicates, the correlated phenotypes, and the multicollinearity of the variants. Table 1 also gives the PoR identifying significant variants averaged over all causal variants for Q1 and Q2. Overall, the average PoR identifying causal variants was quite low for all methods considered. However, for all the methods considered, collapsing rare variants increased the average PoR with significant causal variants compared to methods that used uncollapsed variants.

**Table 1:** Proportion of 200 replicates identifying causal variants in traits Q1 and Q2

Trait	Gene	Variant	PoR for uncollapsed variants			PoR for collapsed variants (MAF < 0.01 and nonsynonymous)		
			
			TR 10^−2^	TR 10^−7^	SLR 10^−7^	TR 10^−2^	TR 10^−7^	SLR 10^−7^
Q1	*ARNT*	C1S6533	0.245	0.005	0.045	0.31	0.02	0.045
	*KDR*	C4S1877	0	0	0.255	0.16	0.005	0.055
		C4S1884	0.16	0.015	0.065	0.2	0.01	0.065
		C4S1889	0	0	0.255	0.025	0	0.01
	*FLT1*	C13S431	0.475	0.095	0.12	0.495	0.08	0.12
		C13S522	0.745	0.115	0.99	0.78	0.165	0.99
		C13S523	1	0.72	1	1	0.72	1
		C13S524	0.115	0.01	0.39	0.03	0	0.005
	Average PoR		7.9 × 10^−2^	2.5 × 10^−2^	8.2 × 10^−2^	1.6 × 10^−1^	4.8 × 10^−2^	1.1 × 10^−1^

Q2	*VNN1*	C6S5380	0.42	0.01	0.03	0.415	0.025	0.03
	Average PoR		2.2 × 10^−2^	6.3 × 10^−4^	4.9 × 10^−4^	7.2 × 10^−2^	2.7 × 10^−3^	1.8 × 10^−3^

TR, tiled regression. SLR, simple linear regression. PoR, proportion of 200 replicates. For the tiled regression, 10^−2^ and 10^−7^ are the critical values; for the simple linear regression, 10^−7^ is the significance level.

Determining a proxy for the type I error was more problematic because there are two different null hypotheses that depend on the amount of phenotypic and genotypic correlation. Table 2 presents the PoR identifying a noncausal variant, averaged over all the noncausal variants that were neither located in the same gene with a causal variant nor correlated with any causal variant (correlation greater than 0.7 in absolute value). These qualifications were necessary to avoid including variants that were in linkage disequilibrium or highly correlated with a causal variant. We used significance levels of 10^−7^ for the SLR and critical values of 10^−2^ and 10^−7^ for the tiled regression. Results are presented for uncollapsed and collapsed variants for traits Q1, Q2, and Q4. In general, the average PoR for the uncollapsed variants had roughly the same magnitude as the average PoR for the collapsed variants. However, what was particularly striking was that the variation in the average PoR for noncausal variants using SLR varied by about three orders of magnitude across traits, ranging from 10^−7^ to 10^−3^ for uncollapsed variants and from 0 to 10^−3^ for collapsed variants. The average PoR for noncausal variants was stable across traits for both critical levels for the tiled regression method with both uncollapsed and collapsed variants. Although the PoRs were stable, too many significant noncausal variants were identified when the tiled regression critical level was 10^−7^ (about 10^−6^), and too few noncausal variants were identified when the critical level was 10^−2^ (about 10^−3^). Although critical values were compared to regression *p*-values for variants included in the final tiled regression model, these values cannot be assumed to represent a significance level for the entire tiled regression procedure. Appropriate significance levels for this procedure are currently being investigated.

**Table 2:** Average proportion of 200 replicates identifying noncausal variants in traits Q1, Q2, and Q4

Trait	PoR for uncollapsed variants			PoR for collapsed variants (MAF < 0.01 and nonsynonymous)		
	
	TR 10^−2^	TR 10^−7^	SLR 10^−7^	TR 10^−2^	TR 10^−7^	SLR 10^−7^
Q1	9.7 × 10^−4^	5.5 × 10^−6^	6.8 × 10^−4^	1.3 × 10^−3^	7.3 × 10^−6^	1.0 × 10^−3^
Q2	1.3 × 10^−3^	6.2 × 10^−6^	3.1 × 10^−6^	1.7 × 10^−3^	3.8 × 10^−6^	2.1 × 10^−6^
Q4	1.3 × 10^−3^	3.5 × 10^−6^	2.0 × 10^−7^	1.7 × 10^−3^	3.2 × 10^−6^	0.0

TR, tiled regression. SLR, simple linear regression. PoR, proportion of 200 replicates. For the tiled regression, 10^−2^ and 10^−7^ are the critical values; for the simple linear regression, 10^−7^ is the significance level.

## Discussion and conclusions

Regardless of the method used, collapsing rare variants generally increased the proportion of replicates that identified a significant causal variant. However, the allele frequency and effect size of the allele for most of the causal variants were too small to be detected in these data using these methods. For Q1, only variants in *FLT1* had a PoR greater than about 0.5. For Q2, only one variant in *VNN1* had a PoR of any sizable magnitude. Nearly all the other causal variants for Q1 and Q2 were undetectable.

More troubling was the inconsistency in the average PoR that identified significant noncausal variants for the SLR method. A difference of about three orders of magnitude was seen for traditional SLR methods, most likely caused by differences in the underlying simulation models for the traits, identical genotypes across replicates, correlated phenotypes, and, perhaps most important, the high degree of multicollinearity in the genotyping data.

The lack of agreement between the empirically derived average PoR and the expected significance levels for the SLR methods may be due to the alternative null hypothesis problem. Under the null hypothesis of no genetic component, no causative variants influence the phenotype; that is, the phenotype is essentially a normally distributed random variable. Under the other null hypothesis, there are no causative variants among the set of variants considered; however, other unknown causal variants do, in fact, influence the phenotypic distribution. These unknown causative variants affect the correlation structure of the phenotypes and may be correlated with other known or unknown variants. Trait Q4 was not generated by specific causative variants but rather is a polygenic effect, which is essentially a normally distributed random variable in unrelated individuals. On the other hand, traits Q1 and Q2 were generated by known causative variants that were correlated with other noncausative variants [8]. These correlations can increase both the type I error rate and the power of the test. This may explain the inflated type I error rate for the SLR methods. The increase in type I error rate for Q1 is quite large, perhaps reflecting the presence of causative variants with relatively larger locus-specific heritability relative to the causative variants in Q2. The tiled regression approach attempts to minimize these correlations by identifying the set of independent variants that most affect phenotypic variation while minimizing the degree of multicollinearity. It appears from these results that the type I error rates for tiled regression are stable with respect to the underlying null hypothesis, although additional work will be required to determine accurate significance levels for the entire tiled regression procedure, not just the significance levels in the final model.

## Competing interests

The authors declare that there are no competing interests.

## Authors’ contributions

AFW and JEBW conceived and designed the study. Analyses were performed by HS, YK, CDC, CLS, QL, BCP and AJMS. AFW, AJMS, JC, HS and YK developed the tiled regression method and software. Data management and programming support was provided by BCP, QL, CLS and AJMS. HS wrote the first, and AFW re-wrote the final drafts of the manuscript. All authors contributed to, and helped to edit the final drafts of the manuscript. All authors read and approved the final manuscript.
